# Impact of Updated NCCN Guidelines on Clinical Management and Risk Communication for *CHEK2* p.I157T Carriers in Breast Cancer

**DOI:** 10.21203/rs.3.rs-8833709/v1

**Published:** 2026-02-22

**Authors:** Emma A. Kell, Michael P. Mullane, Jennifer L. Geurts, Brenda Ramczyk, Tessa M. Bachinski

**Affiliations:** 1. The Medical College of Wisconsin, Milwaukee, Wisconsin; 2. Advocate Health Hereditary Cancer Center, Wisconsin

**Keywords:** CHEK2, hereditary cancer, breast cancer, reduced penetrance, risk management

## Abstract

In 2023, the National Comprehensive Cancer Network^®^ (NCCN^®^) updated its guidelines for managing breast cancer risk in patients with a *CHEK2* p.I157T variant, recommending de-escalation of enhanced screening based on this variant alone. This shift reflects evidence that some missense variants carry a lower cancer risk than *CHEK2* loss-of-function variants. A reduced cancer risk is particularly well established for the *CHEK2* p.I157T variant, prompting the updated guidelines. This study examines healthcare providers’ understanding of these updated guidelines and explores how providers communicate these changes to patients affected by hereditary cancer risks. We conducted a survey targeting healthcare providers involved in hereditary cancer management, capturing their perceptions of the clinical implications of *CHEK2* p.I157T and the approaches used to communicate de-escalated management recommendations. Additionally, a retrospective records review was performed to approximate the percentage of patients impacted by these de-escalation recommendations. This research aims to evaluate factors shaping provider recommendations and communication following guideline changes in hereditary cancer care. Results may inform best practice considerations for patient-provider communication in hereditary cancer care, particularly when new evidence leads to modified screening or management recommendations.

## INTRODUCTION

The NCCN Clinical Practice Guidelines in Oncology (NCCN Guidelines^®^) are a comprehensive set of guidelines detailing the management decisions and interventions through evidence-based, consensus-driven management to ensure that all patients receive preventive, diagnostic, treatment, and supportive services that are most likely to lead to optimal outcomes [1]. For hereditary cancers, including breast cancer, these guidelines are critical for defining best practices in risk assessment and risk management for individuals with inherited cancer predispositions.

Early studies demonstrating that *CHEK2* was associated with increased risk of female breast cancer were based on the truncating European founder variant, c.1100delC (p.Thr367fs, rs555607708) which showed the relative risk for breast cancer to be around two-fold [2, 3, 4]. Risks associated with the *CHEK2 c*.1100delC variant surpass the lifetime cumulative breast cancer risk of >20%, which meets the threshold to recommend high-risk breast cancer screening through annual MRIs in addition to annual mammography with breast imaging offset by six months [5]. In contrast to the typical moderate cancer risks attributed to *CHEK2* pathogenic variants like c.1100delC, another common European founder variant, p.I157T (c.470T>C, rs17879961), imparts a lifetime breast cancer risk of <20% and a relative risk of 1.3, which does not reach thresholds to warrant high-risk breast cancer screening [4, 5, 6].

In 2023, the NCCN revised its guidelines for breast cancer risk management in patients with a *CHEK2* p.I157T variant. The updated NCCN Guidelines^®^ state that this specific variant, in isolation, does not warrant increased screening measures [7]. This revision de-escalates recommendations from prior guidelines, which included high-risk breast cancer screening but acknowledged that missense variants, such as p.I157T, were likely associated with lower cancer risks [8]. This update reflects the current understanding that *CHEK2* p.I157T, a missense variant, carries a lower cancer risk than *CHEK2* truncating variants, such as c.1100delC [4, 9, 10]. Heterozygous truncating variants have a definite association for breast cancer predisposition and a moderate risk for prostate cancer, while prior associations for colorectal cancer risk have been revised in recent guidelines, indicating no increased risk. In contrast, heterozygous missense variants, specifically p.I157T, show that cancer risk and penetrance are below the accepted levels of clinical actionability in isolation [6, 11, 12, 13]. Following these changes, the recommended clinical management of *CHEK2* p.I157T has shifted, presenting new considerations for both providers and patients.

Checkpoint kinase 2 (CHEK2) functions as a tumor suppressor activated in response to DNA damage, and loss of function variants in this gene are associated with moderate cancer risks, particularly for breast and prostate cancers [14, 15]. The broad utilization of next-generation sequencing has increased the frequency at which lower-risk variants like *CHEK2* p.I157T are identified in clinical practice [16]. However, given the variability in risk between missense and truncating variants, clinical guidelines like those provided by the NCCN must continuously adapt to emerging evidence. Furthermore, the NCCN emphasizes that data supporting recommendations for certain genes, including *CHEK2*, are evolving at this time. It advises caution when implementing surveillance regimes, such as colonoscopies following recent changes to the colorectal cancer risk assessment for *CHEK2* and encourages consideration of patient preferences and new knowledge that may emerge [17]. Although these guidelines help standardize care, they also require providers to interpret and apply them within the nuanced context of each patient’s individual risk profile considering the influence of the specific variant, family history, and modifying genetic and nongenetic risk factors, especially as research evolves [1, 12].

Despite the benefits of guideline updates, communicating changes in risk management poses challenges, especially in cases of de-escalation. Revising cancer risk assessments and management can have psychological and emotional impact on patients, and research shows that communication in these contexts is complex, often requiring a shared approach among clinicians, laboratories, and patients [18]. However, as the frequency of reclassifications and changes in recommended management increase, the issue of whether, how, when, and which providers should communicate this information to patients becomes more important and less clear [19]. Additionally, providers are faced with the challenge of understanding the potential clinical implications if reclassification or de-escalation of a variant occurs [20]. These complexities are further compounded by patient risk perception and the role of worry in decision-making, as patients may struggle to reconcile evolving recommendations with personal fears and previous understandings of their risk. This study aims to assess how healthcare providers have understood and adapted to the recent NCCN *CHEK2* p.I157T guideline changes, considering clinic management and how these changes are communicated to patients. Findings from this research could guide future considerations for providers communicating evolving risk management guidelines in hereditary cancer care.

## METHODS

### Survey

Practicing healthcare providers with experience managing hereditary cancer risk were recruited and were invited to complete a one-time survey. The survey was a quantitative assessment of providers’ practice demographics, clinical knowledge of *CHEK2*-associated cancer risks, management practices for *CHEK2*-associated breast cancer risk, and strategies for communicating guideline updates to patients, including provider confidence and methods used in these communications. Survey included up to 24 multiple choice, select all that apply, or free response questions. Recruitment emails were distributed through the National Society of Genetic Counselors (NSGC) and City of Hope and the Clinical Cancer Genomics Community of Practice (CCGCoP) over a 12-week period.

Survey responses were analyzed, with categorical response questions summarized by frequency and percentage. Likert scale responses were summarized both by frequency and percentage for each level, and by the mean and standard deviation on a numeric scale. Select categorical variables were each compared between groups using a Fisher’s exact test. Ordinal categorical variables were compared between groups using a Wilcoxon rank-sum test. All statistical analyses were performed using R version 4.4.1 (2024-06-14 ucrt). All tests were two-sided and *p* < 0.05 was considered statistically significant.

### Chart Review

A retrospective, descriptive chart review of electronic medical and administrative records for patients with a *CHEK2* p.I157T variant seen at the Wisconsin Advocate Health Hereditary Cancer Center (HCC) between 2015 and 2024 was conducted. Patient records were reviewed for demographic information, personal and family cancer history, clinical and genetic risk factors, reproductive and hormone use history, anatomical and physiological characteristics, and documented clinical management decisions.

**Fig. 1:**
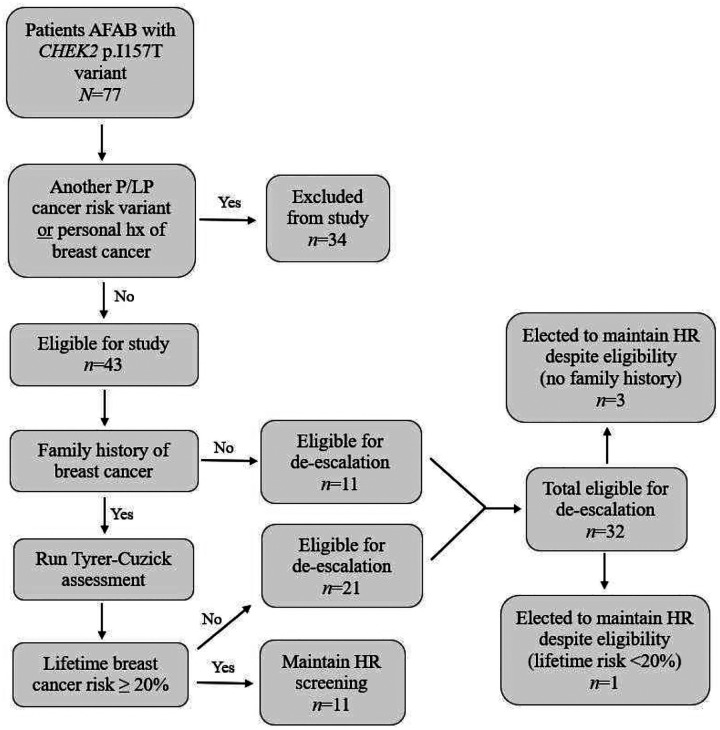
Patient screening distribution following application of prespecified eligibility criteria AFAB: Assigned female at birth, P/LP: Pathogenic or likely pathogenic, HR: High-risk screening

Variables were analyzed for each patient who met the inclusion criteria, including being assigned female at birth, having female breast tissue, carrying a *CHEK2* p.I157T variant, being 18 years or older, and having been seen at least once in an AAH Hereditary Cancer Center. Patients were excluded if they had a personal history of breast cancer, carried an additional pathogenic or likely pathogenic cancer-risk variant, or were deceased. Eligibility for de-escalation was determined based on personal or family history of breast cancer, as illustrated in the flow chart with results of this review in Fig. 1. Patients with no personal or family history of breast cancer qualified for de-escalation. For those with a family history but no personal history, Tyrer Cuzick risk assessment was performed; if the calculated risk was ≥20%, the patient was not considered eligible for de-escalation. If great aunts were affected with breast cancer, they were included in the Tyrer-Cuzick assessment as first cousins, as both are third-degree relatives. Collectively, these data were used to approximate the distribution of patients with a *CHEK2* p.I157T variant in the HCC population across three groups: those whose management would be altered by the updated recommendations, those who still qualified for screening based on personal or family history factors, and those who qualified for de-escalation but elected to continue high-risk breast cancer screening. Variables were analyzed, and groups were summarized by frequency and percentage to characterize patient management decisions.

## RESULTS

### Survey

A total of 151 completed survey responses were collected over the 12-week period. Participants represented practices from 37 unique U.S. states and Washington, D.C. Most respondents identified their role in patient care as genetic counselors (*n*=108, 72%), with additional representation from nurse practitioners (*n*=17, 11%), nurse specialists (*n*=11, 7.3%), physician assistants (*n*=2, 1.3%), and physicians, including medical oncologists (*n*=6) and surgical oncologists (*n*=1), among others. Years of experience in their role ranged from less than one year to more than 25 years, with the majority of participants reporting 2–5 years (*n*=62, 41%) or 6–10 years (*n*=32, 21%) of experience. Experience in hereditary cancer management followed a similar pattern, with most participants reporting 2–5 years (*n*=65, 43%) or 6–10 years (*n*=30, 20%), and few reporting no specific experience in hereditary cancer management (*n*=5, 3.3%). Participants most commonly practiced in hospital-based settings (*n*=55, 36%) or combined academic and hospital settings (*n*=51, 34%), and most reported oncology as their primary practice specialty (*n*=116, 77%).

#### Clinical Knowledge and Practice Application

Of the 151 participants who completed the survey, 89% (*n*=135) reported having previously managed the care for at least one *CHEK2* p.I157T carrier and 91% (*n*=138) reported awareness of the 2023 update to NCCN Guidelines for breast cancer screening for *CHEK2* p.I157T carriers.

The majority (*n*=142, 94%) of survey participants recognized a greater risk for female breast cancer for a *CHEK2* c.1100delC variant than for a *CHEK2* p.I157T variant. A smaller number of participants reported their understanding to be the p.I157T variant conveys clinically significant risk for breast cancer that is greater than the risk for c.1100delC (*n*=6, 4%) or that both variants convey equal risk for breast cancer (*n*=3, 2%). Most (*n*=109, 72%) of respondents indicated that they manage unaffected *CHEK2* c.1100delC carriers for their risk of breast cancer through some form of high-risk screening or surgical measure. Notably, 28% of respondents reported making management decisions for unaffected c.1100delC carriers based on a patient’s personal and family history, incorporating risk modeling tools like Tyrer-Cuzick or CanRisk to aid in assessment (Table 1).

In contrast, the majority (*n*=126, 83%) of providers reported that they manage unaffected *CHEK2* p.I157T carriers through an assessment of personal and family history aided by Tyrer-Cuzick or CanRisk. Fewer (*n*=18, 11.7%) providers indicated managing unaffected p.I157T carriers through some form of high-risk screening management or surgical measure. A smaller subset (*n*=7, 4.6%) of providers reported that they utilize population screening guidelines for unaffected p.I157T carriers (Table 1).

Providers were asked to indicate their understanding of the cancer risks associated with clinically significant *CHEK2* variants, with the option to select all relevant risks. All participants acknowledged an increased risk for breast cancer and 54% recognized an elevated risk for prostate cancer. Additionally, providers identified several other cancer types historically linked to *CHEK2* or with emerging evidence of associated risk (Table S1). Interestingly, providers who reported cancer risks that are not currently well-established or previously associated with *CHEK2*, such as gastric, pancreatic, endometrial, and ovarian cancer, were more likely to have been in practice longer (*p*= 0.02) and to utilize high-risk screening or surgical measures for p.I157T carriers regardless of patient’s personal or family history (*p*= 0.001) (Table 2).

#### Communication of Changes

Providers varied in whether the change in NCCN Guidelines recommending de-escalation of breast cancer screening has been communicated in their practice. More than half of participants (*n*=86, 57%) of providers have personally communicated this change in recommendations, while 13% (*n*=20) of providers reported that they have not personally communicated the change but another provider in their practice has. The remaining 30% (*n*=45) of providers reported that they have never personally communicated this change and were not aware of another provider in their practice having done so. Of the providers who have personally communicated this change in recommendations, 49% (*n*=42) of them reported having communicated this change to approximately 2–5 patients, but responses varied from having communicated the change to a single patient to greater than 50 patients (Table S2).

For practices that have communicated this change to patients, providers indicated that most commonly the change in NCCN Guidelines recommending a de-escalation in breast cancer screening for *CHEK2* p.I157T carriers is communicated verbally at a patient’s appointment (*n*=33, 31%) in their practice and most often disclosed by a genetic counselor (*n*=73, 69%), medical oncologist (*n*=9, 8.5%), or nurse practitioner (*n*=8, 7.5%)(Table S2). Unexpectedly, 39% (*n*=41) of these providers electively shared through a free text response that their practice does not proactively contact patients to share this de-escalation, and it is only communicated if a patient initiates contact regarding *CHEK2* p.I157T status. Given that this question was not directly asked of survey participants, it is possible that an even greater percentage of practices not represented through these data do not proactively recontact patients to assess and share the possibility of de-escalation.

When asked about their comfortability level in communicating the NCCN guideline change for *CHEK2* p.I157T carriers based on adequate information within the NCCN Guidelines, most providers agreed that there is enough information for them to feel comfortable communicating these changes (56% agree, 26% strongly agree; Mean = 1.91, SD = 0.66). Similarly, providers reported high confidence in communicating this change, with 50% feeling “fairly confident” and 34% feeling “extremely confident” (Mean = 1.83, SD = 0.72) (Table S3).

No significant difference in management for *CHEK2* c.1100delC carriers based on provider confidence was noted (*p*=0.218). Importantly, confidence level in communicating guidelines changes did impact management changes for p.I157T. Providers who felt “not very confident” or “not confident at all” communicating guideline changes were more likely to utilize a high-risk screening or surgical measure for p.I157T carriers compared to those who were “extremely confident” (0%) or “fairly confident” (12%) (*p*=<0.001) (Table 3).

### Retrospective Chart Review

The medical records of patients with a *CHEK2* p.I157T variant seen at the Advocate Health Hereditary Cancer Center (HCC) were examined. A total of 77 *CHEK2* p.I157T patient records were considered for review, 43 of these patients met final criteria for study.

Based on the reviewed factors, 32 unaffected patients (74.4%), defined as having no personal history of breast cancer, with a *CHEK2* p.I157T variant in the HCC population would or have had their breast cancer screening management altered by the change in NCCN Guidelines. This conclusion was based on each patient’s family history of breast cancer and/or risk factors assessed using the Tyrer-Cuzick assessment resulting in a score of <20% lifetime risk for breast cancer. Of this subset of patients that qualify for de-escalation of their breast cancer risk screening, 4 patients (12.5%) are known to have elected to maintain previous high-risk breast cancer screening measures as seen in Table 5. Interestingly, three of the four patients who elected to maintain high-risk screening had no family history of breast cancer (Fig. 1).

Eleven unaffected patients (22.6%) with a *CHEK2* p.I157T variant would not or have not had their breast cancer risk management altered by the change in guidelines (Table 4). This conclusion was based on a Tyrer-Cuzick score ≥20% lifetime risk for breast cancer. Although it is possible for a patient to receive a high-risk calculation without a family history of breast cancer, which according to our study’s criteria would result in de-escalation of screening, this did not occur for any patients in our study.

## DISCUSSION

Updates to the NCCN Guidelines for *CHEK2* p.I157T represent a significant shift in the management and risk assessment of hereditary breast cancer. Changes like these will continue to arise and are crucial to improving patient care through evidence-based practice, yet this study shows their implementation has been met with varying degrees of consistency across healthcare providers. This study found that updates to NCCN Guidelines for *CHEK2* p.I157T have impacted clinical management recommendations, influenced risk assessment strategies, and led to variability in provider understanding and application of the new guidelines. Results also highlight challenges in communication of these changes to patients and integrating them into clinical practice.

Consistent with prior studies, our findings highlight that despite existing evidence indicating a lifetime risk for breast cancer to be less than 20%, which does not meet the threshold at which high-risk screening is recommended, there are still inconsistencies in provider management of unaffected *CHEK2* p.I157T carriers [21, 22]. In this study specifically, 11.3% of providers report managing unaffected *CHEK2* p.I157T carriers with some form of high-risk screening or surgical management as their standard recommendation, without assessing personal or family history. The presence of these high-risk management practices potentially represents over-treatment among p.I157T carriers. Other studies report that *CHEK2* germline pathogenic variant (GPV) carrier status is not a significant predictor of cancer risk management practices or treatment uptake, indicating that providers and their patients are not adherent to current GPV-based recommendations. This lack of adherence is further influenced by provider uncertainty surrounding cancer risk estimates and the effectiveness of risk management strategies, which in turn impacts patient uptake of these strategies [5, 23].

It is the responsibility of the practicing provider to stay up to date on current guidelines and evidence-based recommendations, especially in the setting of cancer management. When cancer-predisposition variants are reclassified or existing recommendations are de-escalated, providers should make themselves aware of these changes for consideration in clinical management of their patients. However, providers are faced with the challenge of interpreting updated guidelines and appropriately incorporating these altered recommendations into practice [20]. Numerous factors impact appropriate testing and screening for patients including physicians being unaware of, having low confidence in, or perceived conflict with guidelines [24, 25]. As observed in our study, providers who reported feeling not very confident or not confident at all in communicating the NCCN guideline changes to patients were more likely to inappropriately apply the guidelines in clinical practice, managing unaffected *CHEK2* p.I157T carriers with high-risk screening or surgical measures. Similarly, the 11.7% of providers overall who reported managing unaffected p.I157T carriers with high-risk screening or surgical interventions closely aligns with the 8.6% of providers who were unaware of the 2023 NCCN guideline update for breast cancer risk management in patients with a *CHEK2* p.I157T variant.

Inappropriate applications of evidence-based guidelines may be considered overtreatment of an unaffected individual. Inappropriate applications of guidelines have negative implications for both patients and providers including financial hardship, increased time burden, the potential for overdiagnosis and false positives, emotional burden and a lack of evidence demonstrating clinical utility or improvement in health outcomes [26, 27]. Furthermore, there is economic harm to healthcare systems not limited to resource misallocation, such as increased healthcare expenditures from unnecessary screening tests, treatments, and follow-up visits that are not indicated based on clinical breast cancer risk and have the potential to impact budgetary constraints for federal health insurance programs like Medicare [28].

Revising cancer risk management can have psychological and emotional impacts on patients, and requires shared, sometimes complex decision-making process between clinicians, laboratories, and patients [18, 29]. This process can be further complicated by ambiguous guidelines, patient perception of risk, and provider misinterpretation of guidelines or bias shaped by the circumstances of previous cases. Controversy and uncertainty around recent de-escalation of previous NCCN management guidelines for *CHEK2* p.I157T carriers highlights the need for more studies examining the quality of communication in provider-patient consultations about screening when the guidelines are complicated and controversial [30]. Providers should recognize that their recommendation and the quality and content of discussion around screening management is more nuanced than a simple recommendation. It may have an additional and important bearing on a patient’s decision to get screened even if they qualify for de-escalation of previously recommended screening measures [31]. Studies show that patients value when their provider involves their values, attitudes, and preferences in a shared decision-making conversation about cancer screening. These conversations will continue to be more pronounced as cancer screening decisions become more complex and evolve with the identification of additional etiologic and genetic risk [29, 32, 33].

Given these complexities, it is crucial for providers and their institutions to explore effective strategies for communicating updates to cancer risk management. Ensuring that patients receive updated information about changes to cancer risk management can be challenging, especially when they are seen at a clinic only once for diagnosis and do not have ongoing follow-up. This challenge is reflected in the 41 unprompted provider responses indicating that their clinic does not proactively share the de-escalation of screening for *CHEK2* p.I157T carriers unless the patient initiates contact, despite a desire to do so. Various institutional and logistical barriers, such as high patient volumes and a lack of support staff, limit the ability to recontact these patients. Longitudinal or long-term follow-up clinics, like the Advocate HCC, may be better equipped to initiate these discussions because they see patients repeatedly and their care is significantly guided by known pathogenic variants [34, 35]. As demonstrated by our chart review, a notable proportion of unaffected *CHEK2* p.I157T carriers are potentially impacted by this change in recommended cancer screening, reinforcing the importance of ensuring that genetic testing results are used responsibly to guide appropriate management.

Although our findings regarding patient preference to continue adhering to high-risk screening measures represent a small cohort of people, this underscores the importance of evaluating the impact of guideline changes on patients. Patients may choose to continue with high-risk screening despite eligibility for de-escalation because they fear the possibility of developing cancer due to delayed detection more than they worry about unnecessary care [36]. Importantly, even for patients who express confidence in deciding to continue high-risk screening, they desire help in finalizing their decision from their clinician in receiving information regarding survival benefits, comparative effectiveness of tests, and risks posed by the target cancer [36]. The desire to engage in shared decision-making with a provider stresses the importance of providers being able to adequately address an individual’s cancer risk, be familiar with cancer preventive options, and feel confident with recommendations and delivery [32].

Risk perception by both patients and providers is prone to be influenced by a number of factors including family history, clinical risk scores, and socio-cultural influences or personal health beliefs [37]. This is exemplified by three out of four patients who elected to maintain high-risk screening despite being eligible for de-escalation, even in the absence of a family history of breast cancer. Risk perception is not merely a cognitive process, but an affective and existential one. The relationship between worry and risk perception can influence screening behaviors among patients regardless of whether sound evidence exists for the clinical utility of those screening tests. This creates challenges for practicing clinicians who strive to judiciously utilize tests in a manner consistent with the best available evidence and the patient’s values. Risk perception is unavoidably affectively loaded, and is thus susceptible to manipulation and distortion, making approaches to informed decision-making that acknowledge and work in concert with these influences in high-risk populations, or populations that have previously been managed as high-risk, especially challenging and necessary [38].

## LIMITATIONS

Several limitations should be considered when interpreting the findings of this study. First, the data collected and its interpretation reflect provider knowledge and the application of guidelines at a single point in time. The largest group of participants were genetic counselors (GCs), who are specifically trained in integrating genomic information into patient care. As a result, survey responses from non-GC providers may not fully represent the broader range of provider understanding. Additionally, some nuances in how providers manage breast cancer risk may not have been captured by the response options available in the survey.

The relatively small sample size of reviewed patient records is another limitation. Self-reported family history data, which may be incomplete or inaccurate, could affect risk assessment. The study also reflects the health status of patients from a single healthcare system over a 10-year period, which may limit the generalizability of our findings. Furthermore, the use of the Tyrer-Cuzick assessment for de-escalation of screening may oversimplify risk stratification, as it does not account for all genetic, environmental, or familial malignancy factors that contribute to cancer risk. Finally, as genetic risk information and associated guidelines continue to evolve, the findings of this study may change in relevance as future guidelines are updated.

## FUTURE DIRECTIONS

Future studies should include larger cohorts of survey participants, utilizing additional modalities to ensure a more balanced representation of different provider types involved in cancer risk management. Qualitative methods for assessing provider knowledge and guideline implementation may offer deeper insights into the nuances of management practices and better capture the full spectrum of guideline adherence.

Additionally, future research should explore patient perceptions and experiences regarding the de-escalation of cancer screening when previously managed under high-risk guidelines. The findings from such studies could inform institutional recommendations and procedures for managing the reclassification of cancer risk variants, ensuring that genomic data is used effectively and responsibly.

## CONCLUSION

This study underscores the variability in provider understanding and clinical application of updated NCCN Guidelines for *CHEK2* p.I157T carriers for risk of female breast cancer. These findings highlight the need for ongoing awareness and monitoring of guidelines, particularly for management guided by genetic data as understanding of risk for cancer evolves. Additionally, they highlight the challenges associated with implementing procedures for communicating management changes to patients. Future research should focus on perception of management de-escalation within this patient population and its impact on patient decision-making.

## Figures and Tables

**Table 1:** Provider self-reported clinical management of *CHEK2* variants

Current or theoretical clinical management strategy for unaffected *CHEK2* p.I157T or c.1100delC carrier	*N*=151 (%)
c.1100delC management	
High risk breast cancer screening	101 (67%)
Recommend bilateral mastectomy	1 (0.7%)
Option of high-risk screening or bilateral mastectomy	7 (4.6%)
Population-based screening	0 (0%)
Depends on the patient’s personal and family history. I use additional tools like Tyrer Cuzick or CanRisk to aid in this assessment.	42 (28%)
p.I157T management	
High risk breast cancer screening	17 (11%)
Recommend bilateral mastectomy	0 (0%)
Option of high-risk screening or bilateral mastectomy	1 (0.7%)
Population-based screening	7 (4.6%)
Depends on the patient’s personal and family history. I use additional tools like Tyrer Cuzick or CanRisk to aid in this assessment.	126 (83%)

**Table 2:** Provider understanding of *CHEK2* cancer risks versus years in practice and clinical management

	Selected endometrial, ovarian, pancreatic, and/or gastric cancer as a *CHEK2*-related cancer risk		
Characteristic	No *n*=143^1^	Yes *n*=8^1^	p-value
Years in practice			0.020^2^
1 year or less	18 (13%)	0 (0%)	
2–5 years	61 (43%)	1 (13%)	
6–10 years	29 (20%)	3 (38%)	
11–15 years	14 (9.8%)	1 (13%)	
16–25 years	13 (9.1%)	2 (25%)	
More than 25 years	8 (5.6%)	1 (13%)	
Years in practice (collapsed)			0.026^3^
0–5 Years	79 (55%)	1 (13%)	
6+ Years	64 (45%)	7 (88%)	
*CHEK2* p.I157T management			0.001^3^
High-risk measure	13 (9.1%)	5 (63%)	
No change in medical management	7 (4.9%)	0 (0%)	
Depends on the patient’s personal and family history. I use additional tools like Tyrer Cuzick or CanRisk to aid in this assessment.	123 (86%)	3 (38%)	

1 n (%)

2 Wilcoxon rank sum test with continuity correction

3 Fisher’s exact test

**Table 3:** Provider confidence in communicating change in NCCN Guidelines versus actual management practices

	Confidence in Communicating Change			
Management of Variant	Extremely confident *N*=51^1^	Fairly confident *N*= 76^1^	Not very confident or not confident at all *N*=24^1^	p-value
**c.1100delC**				0.218^2^
High-risk management	41 (80%)	53 (70%)	15 (63%)	
No change in management	0 (0%)	0 (0%)	0 (0%)	
Depends on the patient’s personal and family history. I use additional tools like Tyrer Cuzick or CanRisk to aid in this assessment.	10 (20%)	23 (30%)	9 (38%)	
**p.I157T**				<0.001^2^
High-risk management	0 (0%)	9 (12%)	9 (38%)	
No change in management	5 (9.8%)	2 (2.6%)	0 (0%)	
Depends on the patient’s personal and family history. I use additional tools like Tyrer Cuzick or CanRisk to aid in this assessment.	46 (90%)	65 (86%)	15 (63%)	

1 n (%)

2 Fisher’s exact test

**Table 4:** Screening de-escalation eligibility and patient decisions in unaffected *CHEK2* p.I157T carriers

Clinical management decision	*n*=43^1^
Eligible for breast cancer screening de-escalation^†^	32 (74.4%)
Maintain high-risk (HR) breast cancer screening	11 (25.6%)

† Includes 4 patients (12.5% of de-escalation group) who elected to maintain HR screening despite eligibility for de-escalation.

1 n (%)

## References

[1] National Comprehensive Cancer Network ^®^ (NCCN^®^), “About Clinical Practice Guidelines.” Accessed: Oct. 27, 2024. [Online]. Available: https://www.nccn.org/guidelines/guidelines-process/about-nccn-clinical-practice-guidelines

[2] Meijers-Heijboer H. , “Low-penetrance susceptibility to breast cancer due to CHEK2*1100delC in noncarriers of BRCA1 or BRCA2 mutations: The CHEK2-breast cancer consortium,” Nat. Genet., vol. 31, no. 1, 2002, doi: 10.1038/ng879. 11967536

[3] Breast Cancer Association Consortium, “Breast Cancer Risk Genes — Association Analysis in More than 113,000 Women,” New England Journal of Medicine, 2021, doi: 10.1056/NEJMoa1913948. PMC7611105 33471991

[4] Hu C. , “A Population-Based Study of Genes Previously Implicated in Breast Cancer,” New England Journal of Medicine, vol. 384, no. 5, 2021, doi: 10.1056/nejmoa2005936. PMC8127622 33471974

[5] Garmendia D., Weidner A., Venton L., and Pal T., “Comparing Cancer Risk Management between Females with Truncating CHEK2 1100delC versus Missense CHEK2 I157T Variants,” Genes (Basel)., vol. 15, no. 7, Jul. 2024. 10.3390/genes15070881 PMC11276105 39062660

[6] Kumpula T. A. , “Evaluating the role of CHEK2 p.(Asp438Tyr) allele in inherited breast cancer predisposition,” Fam. Cancer, vol. 22, no. 3, 2023, doi: 10.1007/s10689-023-00327-2. PMC10276058 36653541

[7] Referenced with permission from the NCCN Clinical Practice Guidelines in Oncology (NCCN Guidelines^®^) for Genetic/Familial High-Risk Assessment: Breast, Ovarian, and Pancreatic Version 2.2023. All rights reserved. Accessed March 10, 2025. To view the most recent and complete version of the guideline, go online to NCCN.org.

[8] Referenced with permission from the NCCN Clinical Practice Guidelines in Oncology (NCCN Guidelines^®^) for Genetic/Familial High-Risk Assessment: Breast, Ovarian, and Pancreatic Version 1.2022. All rights reserved. Accessed March 10, 2025. To view the most recent and complete version of the guideline, go online to NCCN.org.

[9] Muranen T. A. , “Patient survival and tumor characteristics associated with CHEK2:p.I157T - findings from the Breast Cancer Association Consortium,” Breast Cancer Research, vol. 18, no. 1, 2016, doi: 10.1186/s13058-016-0758-5. PMC5048645 27716369

[10] Sutcliffe E. G. , “Differences in cancer prevalence among CHEK2 carriers identified via multi-gene panel testing,” Cancer Genet., vol. 246–247, 2020, doi: 10.1016/j.cancergen.2020.07.001. 32805687

[11] Referenced with permission from the NCCN Clinical Practice Guidelines in Oncology (NCCN Guidelines^®^) for Genetic/Familial High-Risk Assessment: Breast, Ovarian, Pancreatic, and Prostate Version 2.2025. All rights reserved. Accessed April 10, 2025. To view the most recent and complete version of the guideline, go online to NCCN.org.

[12] Hanson H. , “Management of individuals with germline pathogenic/likely pathogenic variants in CHEK2: A clinical practice resource of the American College of Medical Genetics and Genomics (ACMG),” Genetics in Medicine, vol. 25, no. 10, 2023, doi: 10.1016/j.gim.2023.100870. PMC10623578 37490054

[13] Mundt E. , “Breast and colorectal cancer risks among over 6,000 CHEK2 pathogenic variant carriers: A comparison of missense versus truncating variants,” Cancer Genet., vol. 278–279, 2023, doi: 10.1016/j.cancergen.2023.10.002. 37839337

[14] Cybulski C. , “CHEK2 is a multiorgan cancer susceptibility gene,” Am. J. Hum. Genet., vol. 75, no. 6, 2004, doi: 10.1086/426403. PMC1182149 15492928

[15] O. Online Mendelian Inheritance in Man and B. M. Johns Hopkins University, “CHECKPOINT KINASE 2; CHEK2,” Online Mendelian Inheritance in Man, OMIM^®^. Accessed: Jun. 01, 2024. [Online]. Available: World Wide Web URL: https://omim.org/

[16] Anaclerio F. , “Clinical usefulness of NGS multi-gene panel testing in hereditary cancer analysis,” Front. Genet., vol. 14, 2023, doi: 10.3389/fgene.2023.1060504. PMC10104445 37065479

[17] Referenced with permission from the NCCN Clinical Practice Guidelines in Oncology (NCCN Guidelines^®^) for Genetic/Familial High-Risk Assessment: Colorectal, Endometrial, and Gastric version 3.2024. All rights reserved. Accessed March 10, 2025. To view the most recent and complete version of the guideline, go online to NCCN.org.

[18] Kilbride M. K., “When variants are reclassified—the importance of personalized communication,” Nat. Med., vol. 26, no. 1, 2020, doi: 10.1038/s41591-019-0707-9. PMC7808556 31932785

[19] Makhnoon S., Davidson E., Shirts B., Arun B., and Shete S., “Practices and Views of US Oncologists and Genetic Counselors Regarding Patient Recontact After Variant Reclassification: Results of a Nationwide Survey,” JCO Precis. Oncol., no. 7, 2023, doi: 10.1200/po.23.00079. PMC10581618 37384863

[20] David K. L. , “Patient re-contact after revision of genomic test results: points to consider—a statement of the American College of Medical Genetics and Genomics (ACMG),” Genetics in Medicine, vol. 21, no. 4, 2019, doi: 10.1038/s41436-018-0391-z. 30578420

[21] Ivanov M. , “Letter to the Editor: CHEK2 I157T - Pluto Among Numerous Low-Risk Genetic Factors Requiring Discharge From a Range of Pathogenic Variants?,” 2022. doi: 10.6004/jnccn.2021.7103. 35130501

[22] Weis L., Bychkovsky B., Rodríguez A. H., Barroso-Sousa R., and Sandoval R., “CHEK2-related breast cancer: real-world challenges,” Fam. Cancer, vol. 24, no. 23, Feb. 2025, doi: 10.1007/s10689-025-00448-w. 39966186

[23] Reyes K. G., Clark C., Gerhart M., Newson A. J., and Ormond K. E., “‘I wish that there was more info’: characterizing the uncertainty experienced by carriers of pathogenic ATM and/or CHEK2 variants,” Fam. Cancer, vol. 21, no. 2, 2022, doi: 10.1007/s10689-021-00251-3. 33855648

[24] Goodwin J. S., Singh A., Reddy N., Riall T. S., and Kuo Y. F., “Overuse of screening colonoscopy in the medicare population,” Arch. Intern. Med., vol. 171, no. 15, 2011, doi: 10.1001/archinternmed.2011.212. PMC3856662 21555653

[25] Predmore Z., Pannikottu J., Sharma R., Tung M., Nothelle S., and Segal J. B., “Factors Associated With the Overuse of Colorectal Cancer Screening: A Systematic Review,” American Journal of Medical Quality, vol. 33, no. 5, 2018, doi: 10.1177/1062860618764302. PMC6404527 29546768

[26] Altice C. K., Banegas M. P., Tucker-Seeley R. D., and Yabroff K. R., “Financial hardships experienced by cancer survivors: A systematic review,” 2017. doi: 10.1093/jnci/djw205. PMC6075571 27754926

[27] Brill J. V., “Screening for cancer: The economic, medical, and psychosocial issues,” American Journal of Managed Care, vol. 26, 2020, doi: 10.37765/AJMC.2020.88534. 33200894

[28] Srivastava S. , “Cancer overdiagnosis: a biological challenge and clinical dilemma,” Nat. Rev. Cancer, vol. 19, no. 6, 2019, doi: 10.1038/s41568-019-0142-8. PMC8819710 31024081

[29] Jimbo M. , “What is lacking in current decision aids on cancer screening?,” CA Cancer J. Clin., vol. 63, no. 3, 2013, doi: 10.3322/caac.21180. PMC3644368 23504675

[30] Alvarado M., Ozanne E., and Esserman L., “Overdiagnosis and Overtreatment of Breast Cancer,” American Society of Clinical Oncology Educational Book, vol. 32, no. 1, May 2012. 10.14694/EdBook_AM.2012.32.301 24451829

[31] Peterson E. B. , “Impact of provider-patient communication on cancer screening adherence: A systematic review,” 2016. doi: 10.1016/j.ypmed.2016.09.034. PMC5518612 27687535

[32] Samimi G. , “Cancer Prevention in Primary Care: Perception of Importance, Recognition of Risk Factors and Prescribing Behaviors,” American Journal of Medicine, vol. 133, no. 6, 2020, doi: 10.1016/j.amjmed.2019.11.017. PMC7293933 31862335

[33] Sepucha K. R., Fowler F. J., and Mulley A. G., “Policy support for patient-centered care: The need for measurable improvements in decision quality,” 2004. doi: 10.1377/hlthaff.var.54. 15471772

[34] Rogen K., Ramczyk B., Bachinski T., Wham D., Schoenebeck A. M., and Mullane M. P., “A Comprehensive Cancer Risk Management Clinic for Families With Hereditary Cancer Syndromes Outcomes After 6 Years,” 2023. doi: 10.3928/25731777-20230710-08.

[35] Knerr S. , “Longitudinal adherence to breast cancer surveillance following cancer genetic testing in an integrated health care system,” Breast Cancer Res. Treat., vol. 201, no. 3, 2023, doi: 10.1007/s10549-023-07007-w. PMC10503958 37470892

[36] Woolf S. H. , “Engaging Patients in Decisions About Cancer Screening: Exploring the Decision Journey Through the Use of a Patient Portal,” Am. J. Prev. Med., vol. 54, no. 2, 2018, doi: 10.1016/j.amepre.2017.10.027. PMC7144024 29241715

[37] Lebrett M. B. , “Risk perception and disease knowledge in attendees of a community-based lung cancer screening programme,” Lung Cancer, vol. 168, 2022, doi: 10.1016/j.lungcan.2022.04.003. 35430354

[38] Tilburt J. C. , “Factors influencing cancer risk perception in high risk populations: A systematic review,” Hered. Cancer Clin. Pract., vol. 9, no. 1, 2011, doi: 10.1186/1897-4287-9-2. PMC3118965 21595959

